# A Scoping Review on Staff Attitudes towards the Use of Coercion in Mental Healthcare

**DOI:** 10.3390/healthcare12161552

**Published:** 2024-08-06

**Authors:** Simone Agnes Efkemann, Jakub Lickiewicz, Paul Doedens, Tella Lantta, Panagiota Bali, Tonje Lossius Husum

**Affiliations:** 1 Department of Psychiatry, Psychotherapy and Preventive Medicine, LWL University Hospital, Ruhr University Bochum, 44791 Bochum, Germany; 2 Department of Health Psychology, Jagiellonian University Medical College, 31-501 Krakow, Poland; 3 Department of Psychiatry, Amsterdam University Medical Center, 1105 AZ Amsterdam, The Netherlands; 4 Urban Vitality—Centre of Expertise, Faculty of Health, Amsterdam University of Applied Sciences, 1105 BD Amsterdam, The Netherlands; 5 Department of Nursing Science, University of Turku, 20014 Turku, Finland; tella.lantta@utu.fi; 6 Centre for Forensic Behavioural Sciences, Swinburne University of Technology, Melbourne 3122, Australia; 7 Second Department of Psychiatry, Medical School, University General Hospital “Attikon”, National and Kapodistrian University of Athens, 12462 Chaidari, Greece; 8 Faculty of Health Sciences, Oslo Metropolitan University, 0130 Oslo, Norway

**Keywords:** attitude of health personnel, psychiatry, mental health services, coercion, social psychology, SACS

## Abstract

Attitudes of mental health professionals towards the use of coercion are highly relevant concerning its use coercion in mental healthcare, as mental health professionals have to weigh ethical arguments and decide within a legal frame in which situations to use coercion or not. Therefore, assessment of those attitudes is relevant for research in this field. A vital instrument to measure those attitudes towards the use of coercion is the Staff Attitude to Coercion Scale. This scoping review aims to provide a structured overview of the advantages and limitations in the assessment of attitudes toward coercion. We conducted a scoping review in Medline, PsycINFO, CINAHL, and Web of Science, based on the PRISMA-ScR. Inclusion criteria were empirical studies on the attitudes of mental health professionals. We included 80 studies and systematically mapped data about the main results and limitations in assessing attitudes toward coercion. The main results highlighted the relevance and increased interest in staff attitudes towards coercion in mental healthcare. Still, the majority of the included studies relied on a variety of different concepts and definitions concerning attitudes. The data further indicated difficulties in developing new and adapting existing assessment instruments because of the equivocal definitions of underlying concepts. To improve the research and knowledge in this area, future studies should be based on solid theoretical foundations. We identified the need for methodological changes and standardized procedures that take into account existing evidence from attitude research in social psychology, nursing science, and other relevant research fields. This would include an update of the Staff Attitude to Coercion Scale based on the limitations identified in this review.

## 1. Introduction

Attitudes play an essential role in human behavior. They support planning and decision-making processes and allow us to obtain a sensible and orderly perception of the world. Attitudes are a framework for organizing and simplifying complex and ambivalent information from the environment and are important for a person to adequately understand information [1]. A common definition of attitudes defines it as the summary evaluation of a specific subject. Furthermore, attitudes can be summarized in the Tripartite model, meaning that attitudes have three components: affective (e.g., emotional-evaluative and emotional-motivational), cognitive (e.g., knowledge, beliefs, opinions, messages, suspicions, doubts, and thoughts), and behavioral (action, motivation, tendency to act, and tendency to behavior) [2].

Professionals’ attitudes are essential in their decision-making, especially in high-demanding situations. An example of such a situation is the use of coercive measures in mental healthcare. In terms of the above-mentioned definition of attitudes in general, attitudes toward coercion can be defined as the summary evaluation of the use of coercion. According to Ajzen and Schmidt, any action (such as the decision to use coercive measures) is the result of social influence and personal factors. Social factors are perceived as social pressures that lead to conducting or omitting a given act (subjective norms) [3]. Understanding other people’s expectations, the value of certain behaviors, and the tendency to comply with other people’s expectations (motivation to comply) are important characteristics of social influence. Subjective norms affect peoples’ intentions and lead to motivation, which is necessary to engage in certain behaviors [4,5]. This leads to intentions based on attitudes about certain behaviors, as well as norms and perceived control over those behaviors [5]. Attitudes resulting from beliefs about behavior and its potential consequences strongly influence peoples’ intentions [3,6]. Furthermore, according to the theory of planned behavior (TPB), positive attitudes and positive subjective norms lead to the increased performance of a give activity, while this effect is moderated by the perceived control of the respective behavior [6]. Subsequently, the attitudes of professionals towards patient behavior and coercive measures highly affect the subjective norms they hold and, thereby, the perceived appropriateness of a coercive measure in given circumstances.

Coercion in mental health care is associated with acting against the will of a patient and therefore is connected with several ethical and legal dilemmas. When using coercive measures, professionals deal with ethical and legal dilemmas. For example, coercive measures are, from a human rights perspective, a violation of patients’ autonomy and physical integrity, but professionals can view such measures as essential to their safety [7]. Personal perceptions and beliefs play a significant role in the decision-making process regarding the use of coercion [8], and professionals’ attitudes can influence the treatment process [9,10]. The lack of knowledge about the influence of attitudes and emotional factors may undermine the decision-making process concerning coercive measures. Because of their essential role in decision-making around coercive measures, attitudes are the subject of research with an increasing evidence base [11,12]. Several authors consider the role of attitudes as an important factor in interventions aiming to reduce the use of coercion in mental healthcare [13,14]. Subsequently, to accomplish a mindset change in mental healthcare, it is essential that staff training focuses on influencing staff attitudes towards coercion [12,15].

Validated questionnaires are available to measure staff attitudes towards coercion in mental healthcare. One of the first tools to explore this phenomenon was a questionnaire by Klinge, investigating the attitudes and opinions of the staff regarding physical restraint and seclusion of patients [16]. A more recent questionnaire is the Attitudes to Containment Measures Questionnaire (ACMQ), developed by Bowers and colleagues [17,18], which measures how staff attitudes rate whether different forms of coercive measures are justifiable. Another approach for assessing attitudes toward coercion is the Knowledge, Attitudes, and Practice (KAP) model [19,20]. Furthermore, the Implicit Association Test (IAT) or the Go/No-Go Association Task (GNAT) are available to assess attitudes and beliefs that people may be unwilling or unable to report [21,22]. IAT has lately been used to provide insights into the relationship between implicit attitudes to the use of coercion and actual behavior regarding coercion in mental healthcare, as well as between implicit and explicit attitudes to the use of coercion [23]. A recently published review has furthermore evaluated the psychometric properties of research instruments for the assessment of attitudes of mental healthcare professionals towards the use of restraint. Though the authors identified a variety of instruments, one of the two most used instrument was the Staff Attitude to Coercion Scale (SACS). In this review, the SACS was the only instrument that allowed a review of psychometric properties and, in this context, was identified as the instrument that has been most validated in previous studies [24]. Furthermore, the SACS represents the only instrument that addresses attitudes to coercion in general without focusing on a specific coercive measure. This seems relevant in terms of comparing attitudes in different settings or countries that use different coercive measures.

The SACS is a widely used and effective tool for measuring staff attitudes towards coercion in mental healthcare, developed in Norway [25]. This 15-item tool aims to assess the attitudes of psychiatric institution personnel toward the use of coercion, using a 5-point Likert scale. The questionnaire asks the subject about their view on coercion in treatment and is explicit that no answer is right or wrong. The developmental study of SACS identified three types of attitudes: (1) coercion as offending to the patient (critical attitude)—the view that coercion violates the patient’s subjectivity; (2) coercion as a care and security (pragmatic attitude)—the view that coercion is needed for care and safety purposes; and (3) coercion as a form of treatment (positive attitude)—the view that coercion may constitute a therapeutic intervention. The original version has adequate psychometric properties [25]. Several authors have translated the SACS into their own language, indicating its potential for cross-cultural applicability [26,27,28,29]. A current review considering the psychometric properties of the SACS in existing studies has summarized the internal consistency for the total score ranging between 0.58 and 0.84, with most studies reporting a score higher than 0.70. A comparable result was found for the individual subscales [30].

In comparison to other tools, the SACS is the only validated questionnaire that measures the general attitudes of mental health professionals toward the use of coercion in psychiatry, without focusing on specific forms of coercive measures. The questionnaire proved its use in various populations, including patients and caregivers. The SACS measures only explicit attitudes towards coercion and has been used in previous studies to examine associations between those attitudes and the incidence of coercive measures [30,31,32]. Additionally, some studies aimed to measure factors influencing attitudes and assess differences between wards in staff attitudes towards coercion [31,33].

Nonetheless, results from existing studies highlight the limitations of the SACS and other instruments in assessing staff attitudes towards coercion [24,31]. Therefore, the objective of our study was to systematically map these limitations of the SACS and the assessment of staff attitudes towards coercion in general. In doing so, we aimed to provide a structured overview of those aspects to facilitate the future development of instruments and research in this area. For this, we conducted a scoping review with the underlying research questions: (1) What do we know from research about staff attitudes towards coercion in mental healthcare since the SACS was developed? (2) Which challenges in the assessment of attitudes towards coercion can be identified from previous studies? (3) Which implications do they raise regarding the update of the SACS?

## 2. Methods

### 2.1. Design

We conducted a scoping review according to the Preferred Reporting Items for Systematic Reviews and Meta-Analyses for Scoping Reviews (PRISMA-ScR). In comparison to a previously conducted review [31], in the present review, we specifically addressed relevant aspects for adaptation or update of the SACS and focused on studies that did or did not use the SACS itself. We conducted the literature search according to a study protocol that has previously been registered in INPLASY (INPLASY202390007).

### 2.2. Eligibility Criteria

We included qualitative and quantitative empirical studies that addressed the attitudes of professionals in mental healthcare towards the use of coercive measures. Based on pilot testing in the first search results, we defined our inclusion criteria as follows: (1) study population included mental health professionals and staff members; (2) intervention or study objective included attitudes towards coercion, normative attitudes, and feelings/emotions and excluded decision-making, experiences, and ethical challenges as those do not fall under the scope of the definition of attitudes as provided in the introduction; and (3) study characteristics included empirical studies, adult psychiatry setting, and outpatient setting (not online). Our exclusion criteria were as follows: (1) service users, informal carers, and students as study participants; (2) adolescent or juvenile settings, forensic or prison settings, and elderly care settings; (3) literature reviews, editorials, comments, letters, thesis, and book chapters; (4) Studies published before 2008, as the original version of the SACS was published then. Inclusion was independent of whether the respective study design or research question specifically addressed attitudes toward coercion. We used no exclusion criteria on language. 

### 2.3. Search Strategy

A librarian at Oslo Metropolitan University conducted the literature search, which consisted of combinations of three main search terms/words and their synonyms: coercion, professional working in mental health services, and attitudes. We provide our full search history in the supplement (Supplementary File S1).

### 2.4. Study Selection

The authors performed the study selection using the web-based software Covidence (Covidence systematic review software, Veritas Health Innovation, Melbourne, Australia. Available at www.covidence.org). First, two authors reviewed all titles and abstracts for eligibility based on our inclusion and exclusion criteria. Afterward, we gathered full-text manuscripts of the remaining references, and two authors screened those independently. We solved any disagreement by consensus or based the decision on a third author.

### 2.5. Data Extraction

All authors performed data extraction using Covidence. For each reference, one author conducted the data extraction, and a second author concluded the final extraction and checked for correctness. We extracted the following information from the included studies:

(1) General information including title, lead author, year of publication, and country.
(2) Study characteristics, specifically study aim, study population, study design, the used definition of “attitudes”, assessment instrument, and description of assessment of attitudes.
(3) Main results.
(4) Limitations regarding attitudes and their assessment.

We used SPSS 29 to conduct numerical descriptive analysis for all quantitative variables (year of publication, country of origin, study aim, study population, study design, and assessment instrument used) and thematic analysis using Excel for all qualitative variables (definition of attitudes, main results, and limitations). Qualitative data were grouped and analyzed numerically. Furthermore, we identified different groups in terms of study aims and used these groups as categories in which we qualitatively summarized the respective main results from those studies. In the analysis of study limitations, we focused on the limitations that included studies reported regarding attitudes towards coercion (and the assessment of those attitudes).

## 3. Results

### 3.1. Search Results

**Figure 1:**
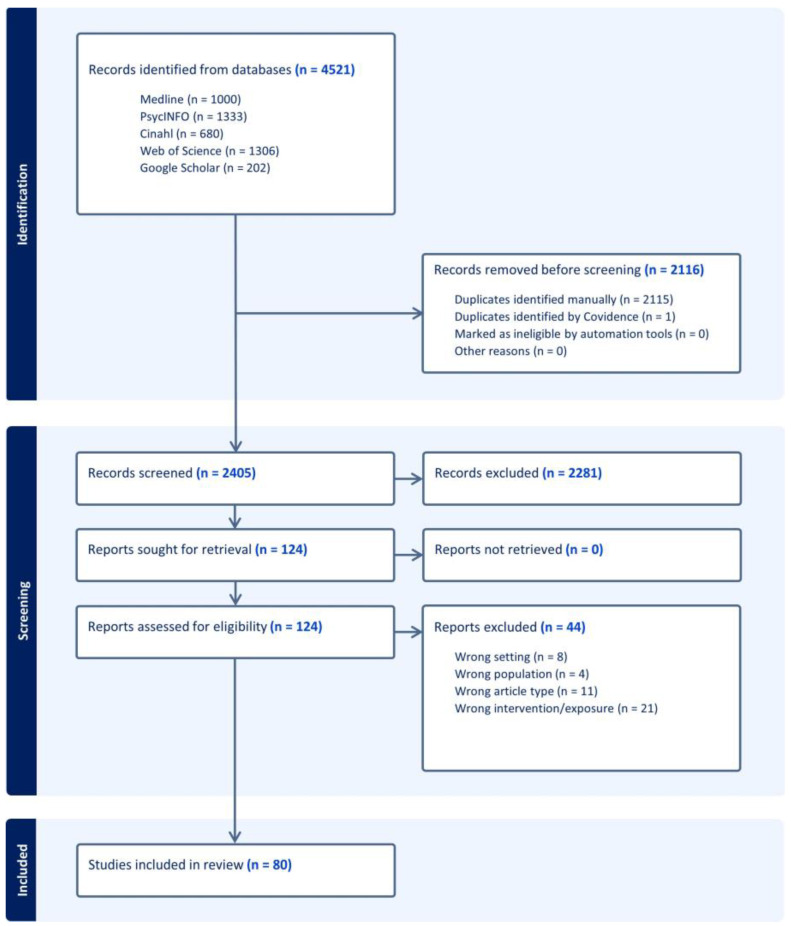
Flow chart of the article selection process according to PRISMA ScR (created by Covidence).

In total, the search resulted in 4521 references. The medical information specialist identified and removed *n* = 2115 duplicates. Therefore, we entered *n* = 2406 references into Covidence, which removed one more duplicate. After the selection of title and abstract, *n* = 124 references remained for full-text assessment. This resulted in *n* = 80 studies included in this scoping review. We summarized the selection process in a PRISMA flow chart (Figure 1).

### 3.2. Aims and Key Findings of Studies Reviewed

Table 1 provides an overview of general information and study characteristics including the study aim. As can be seen there, most of the studies were published in the last five years, stemmed from Asia or Europe, addressed nurses as the study population, and were conducted with a quantitative study design. Furthermore, the studies were quite heterogeneous in their reported study aims. In total, 65 studies explicitly addressed staff attitudes towards coercion in the aim of the study, with differences in the specific research questions. The key findings of the different study aims will be further summarized shortly. We provide a list of all studies including extracted data in terms of general information, study characteristics, and key findings in the Supplementary Materials (Supplementary File S2).

(1) Overall, we found substantial differences in attitudes towards coercion throughout the studies [34,35], especially between different settings, but also between specific types and indications for coercive measures [36,37,38]. The observed studies reported that staff’s perceptions of coercion (including attitudes, emotions, opinions, or general views) were associated with staff characteristics, such as professional background, work experience, age, and gender, but the direction of these associations was highly equivocal [23,27,33,39,40,41,42,43,44,45,46,47,48,49,50]. Attitudes were strongly associated with hospital traditions and policies but also connected with a lack of institutional support [33,51,52]. Studies that reported on emotions related to the use of coercion identified predominantly negative emotions, including helplessness, grief, guilt, and anxiety, but also positive emotions such as feelings of power and control [9,53,54,55]. Further results indicated the relevance of the emotional regulation of staff members in the context of using coercion [56]. In general, staff perception towards coercion seemed to reflect the dilemma of not wanting to use coercion but also perceiving it as necessary, or even beneficial, for the patient in specific situations [54,57,58,59,60,61,62,63].
(2) The studies that addressed the relationship between staff attitudes towards coercion and other individual characteristics reported associations with emotion-focused coping strategies as well as empathy skills [43,64]. Furthermore, attitudes towards coercion were associated with therapeutic optimism, job satisfaction, and emotional exhaustion [65,66,67,68]. Some authors reported differences in attitudes towards coercion between professional groups, specifically in terms of decisional responsibility [69,70]. Critical attitudes towards coercion were especially associated with more optimistic recovery expectations and were more prevalent in staff members working in wards that used less coercive measures, had lower bed occupancy, and had fewer involuntary admissions [71]. The perception of aggression as dysfunctional or undesirable, as well as the staff’s own experience of anger, was associated with the approval of specific coercive measures [72,73].
(3) The results from studies examining the association between staff attitudes to and the actual use of coercion suggest a bidirectional relationship. Several studies reported that a positive attitude towards coercion was associated with more personal involvement in coercive incidents [74,75,76]. More specifically, attitudes differed depending on whether mental health professionals were present during coercive measures or not. Being present during the use of coercion seems to be associated with holding the attitude of coercion representing security and care rather than being offending. Professionals who were not only present during the coercive incident but also physically involved showed even more positive attitudes [77]. Furthermore, other studies found an association between the decision to use coercion and attitudes towards coercion, with a more positive attitude towards coercion increasing the likelihood of deciding to use coercive measures [45,48,58,78]. Beyond attitudes, the decision to use coercion also depends on the knowledge and skill levels of individual staff members [76].
(4) In the comparison of attitudes towards coercion of staff members with those of other relevant groups, differences were found with a higher acceptance of coercive measures in healthcare professionals compared to service users and relatives [51,79,80,81]. When comparing professionals working in different services, greater acceptance and a higher use of coercive measures were found in services that provided access to such measures [82]. Regarding the effect of interventions on staff attitudes, most studies reported significant improvement in attitudes after implementing specific training or interventions to reduce coercion [83,84,85,86,87,88]. Only one study reported no effect of interventions on recovery orientation on attitudes toward coercion [89].
(5) Several studies specifically reported on the translation process of the SACS into different languages, including psychometric validation. These studies mainly confirmed the construct validity of the SACS, though some authors recommended not using the three original subscales, but a total score instead [26,28,29,90]. Two included studies reported specific results on the validation of newly developed questionnaires: one from Germany (Cologne Questionnaire on Attitudes Towards Coercive Measures, KEZ) [91] and one from Israel (“staff attitudes toward coercion use in treatment of mentally ill patients”) [92].

### 3.3. Challenges in the Assessment of Staff Attitudes towards Coercion

Beyond the key findings, we also extracted information on the challenges in the assessment of staff attitudes toward coercion from the studies reviewed. Despite most studies addressing staff attitudes in the aim, only 22 studies provided information on how they defined “attitudes” in their respective study, from which we synthesized different definitions. Altogether, 68 of the studies provided specific information on how they assessed the staff attitudes towards coercion, with *n* 20 using a self-developed questionnaire or interview guideline. The remaining 48 of the studies used at least one originally validated instrument, though most studies used instruments without explicit validation after translation or adaptation for specific study purposes. Furthermore, 33 of the studies reported limitations concerning the assessment of staff attitudes toward coercion. Table 2 provides an overview of definitions of attitudes and assessment instruments and addresses the limitations in the studies reviewed.

A general topic that arose as a limitation was the use of self-reported questionnaires, as several authors discussed that those may limit an in-depth understanding of attitudes and provide poor accuracy [39,43,65,83]. Qualitative studies were helpful in this regard, as they also included an examination of the rationale and thought process behind attitudes [66,83]. In addition, attitudes were often surveyed using a cross-sectional design and, therefore, limit any causal claims about the reported associations. The authors of those studies claimed that experimental studies and longitudinal study designs are necessary [33,37,39,93]. As staff attitudes towards coercion represent a sensitive topic, some studies addressed certain biases of acceptability that might even occur in anonymous, self-reported questionnaires [44,57,94]; there might be an a priori difference between staff members participating in studies regarding attitudes towards coercion and those not willing to participate [51,57]. Most questionnaires only address the cognitive component of attitudes, neglecting the emotional and behavioral components [45,78,89]. Several studies discussed that the existing evidence on the general stability versus the possible fluidity of staff attitudes towards coercion is insufficient. This goes along with reports of missing data on the influence of specific interventions on attitudes [37,81,89].

When adapting instruments regarding attitudes towards coercion, translation might be difficult due to existing differences in local terminology, leading to translation bias [43,95]. Difficulties in the comparison of studies might also arise when studies modify the structure or modus of assessment instruments without sufficient empirical validation, as has been discussed in several studies [9,23,26,32,70]. Furthermore, one study specifically discussed that in the development of new instruments, construct validity cannot be tested against a gold standard due to the lack of other tools so far [29], which is a universal problem for latent constructs such as attitudes. Further reported limitations were regarding generalizability, as many studies have been conducted in highly specific areas (e.g., only in one state or even one hospital) [33,52,66,96,97]. Subsequently, several studies reported difficulties regarding cultural or institutional influence on staff attitudes [59,64,69,90,94,98]. Some studies noted that the attitudes of other relevant stakeholders as well as service users and relatives need addressing, though no validated questionnaires exist in this regard [54,66,74].

## 4. Discussion

The number of studies that investigate the attitudes of mental health staff at towards coercion is varying over time but overall increasing. The relevance of human rights in healthcare has been globally discussed, especially since the publication of the Convention on the Rights of Persons with Disabilities (CRPD) in 2008 [99]. The discussion on human rights has a strong impact also on clinical practice in mental health services and challenges long-time traditions, including the belief in using coercion [100]. According to the CRPD, it must be abolished accordingly as it violates human rights. In a recent call on the need to move away from coercive approaches, they stated that coercion “is incompatible with contemporary human rights principles and standards” [101]. Furthermore, in their guideline on mental health, human rights, and legislation, the WHO also indicated that it is an essential legislative provision to eliminate coercion in mental health services, and upholding the right to free and informed consent should be created [102]. The increasing interest in measuring staff attitudes towards coercion might reflect this worldwide concern, as the use of coercion in mental health challenges the human rights of patients affected [103,104]. This is highlighted by the research and reports of patients’ experiences of the harm caused by coercive measures [105] and also reflected by a strong ethical dilemma experienced by mental health professionals [106]. Along with the discussion on human rights comes the necessity of evidence-based health services, interventions, and treatment [107,108]. A lack of research or ambiguous evidence regarding e.g., community treatment orders [109], raises new challenges for (mental) health professionals. Research on the geographical variation in health services further supports the assumption that lack of evidence for specific services results in doubt and differing beliefs regarding effective interventions in professional narratives [110]. Subsequently, the lack of evidence leaves room for local cultures and the individual beliefs of professionals to guide decision-making. However, variations in the use of coercion, as well as the initiatives to reduce coercion that show positive effects, challenge the belief that coercion is inevitable. This might lead to more extensive efforts to better understand the psychological factors associated with the decision to use coercive measures.

When considering the role of staff attitude in this regard, the association between attitudes and behavior needs further consideration. Evidence supports a bidirectional relationship between attitudes toward coercion and the actual use of coercion in clinical practice. This means that attitudes towards coercion might influence if and how often coercive measures are used, but the use of coercion in clinical practice can also influence the attitudes of mental health professionals towards its use. This is relevant in terms of existing approaches to reduce coercion, as changes in the attitudes of mental health professionals are often viewed as essential in reducing the use of coercive measures or that negative attitudes are a barrier to the reduction of coercion [111,112,113]. Furthermore, attitudes may also influence the intention to implement interventions to reduce the use of coercive measures. Especially in such an ethically controversial topic, it is likely that mental health professionals experience a high degree of moral doubt and cognitive dissonance when they have to use coercion in clinical practice [69,100,114,115]. This might result in positive attitudes towards coercion to cope with such cognitive dissonance. Furthermore, interventions aiming to reduce coercion or educating mental health professionals in this direction might also lead to cognitive resistance preventing any relevant changes in this regard [116,117,118]. Therefore, when implementing measures to reduce coercion, specific additional interventions should be conducted to address mental health professionals’ attitudes, including, specifically, the handling of arising cognitive dissonance. To do this, mental health services need specific interventions addressing staff attitudes towards coercion. The evidence reviewed in this paper provide promising insights, but several theoretical questions still need addressing before the development of specific interventions is feasible. One main question in this context is the stability of staff attitudes towards coercion. Most of the reviewed studies used a cross-sectional design and did not assess attitudes toward coercion in a longitudinal design. This might be especially relevant considering the variety of factors that are associated with staff attitudes towards coercion, e.g., age or professional experience. This might also reflect or be explained by a general change in attitudes over time [119,120]. Many of the studies reviewed discussed the influence of cultural background on staff attitudes towards coercion, which would indicate a lower stability of those attitudes than expected. This suggests opportunities to change those attitudes in a favorable direction with specific interventions, keeping in mind that those might conflict with the reality of clinical practice. Nevertheless, the included studies in this review found little evidence of the effects of such training or interventions on staff attitudes towards coercion. Furthermore, the influence of cultural background on attitudes needs consideration, which also includes organizational culture toward treatment philosophy [35,83,92,97]. As a practical implication, mental health care workers should nevertheless be encouraged to make themselves aware of existing attitudes and to critically reflect on how those might influence their clinical practice.

Considering the relevance of attitudes towards coercion for the reduction of coercive measures, studies that address attitudes need solid theoretical foundations. The evidence included in this review show a substantial variety of concepts and definitions of attitudes. In social psychology, attitude is a frequently studied concept, and results from this area should be applied more strictly to the phenomenon of coercion in mental healthcare. This is especially relevant regarding the different underlying concepts of attitudes in existing studies, raising the challenge to identify the most suitable concept and explain how attitudes towards coercion might influence the actual use of coercive measures. Depending on the research question, attitudes might be defined in terms of (moral) values and normative beliefs [121] or in terms of the three components (cognitive, emotional, and behavioral) [2]. The choice of the definition used needs a clear explanation in such studies. As stated, the use of coercive measures is ethically a highly controversial practice. Therefore, it does not seem reasonable to omit the emotional aspects of attitudes when trying to examine the relationship between attitudes toward coercion and the use of coercive measures. Those aspects are critical for developing new or adapting existing assessment instruments. However, our results revealed several challenges in this regard. When developing new instruments, construct validity cannot be tested against a gold standard due to the lack of other tools. The described variety of differing concepts further challenges this, because, in terms of attitudes toward coercion, we face a latent construct, of which the formal definition is not universally used in all studies. In the adaptation of existing instruments to different languages or settings, the authors identified several biases due to cultural or organizational differences, specifically complicating the translation of respective items.

We based this scoping review on a thorough database search that was not limited by the language of publications. However, unclear terminology could have resulted in the exclusion of relevant studies. In the analysis of study limitations, we did not perform a formal assessment of methodological rigor. Furthermore, our research team was multidisciplinary, providing different perspectives on the examined subject. Nonetheless, the developer of the SACS was part of the review team, and, in addition, further authors of the papers included were involved in the review. We aimed to address this by specifically discussing and following set criteria in cases of conflicts. Furthermore, our general aim was to critically reflect on the SACS instrument and its limitations. 

The considerations from the results raise several implications of concern about updating the SACS, and we suggest an adaptation of it according to the following points: It seems necessary to add items assessing the emotional (and behavioral) component in the questionnaire, as several studies addressed this as a limitation in the current assessment. The German KEZ questionnaire is a good starting point, as the authors did include those components [91]. Furthermore, new versions of the SACS require development and validation to allow the assessment of attitudes towards coercion from other relevant stakeholders, such as service users, relatives, and professionals outside mental healthcare, to enable a valid comparison of the attitudes of those stakeholders. As the cultural background highly influences the attitudes assessed with the SACS, several difficulties arise when translating and adapting the SACS to other countries. To facilitate this process and enable international comparison of attitudes, there is a need for a standard procedure for cultural adaption of the SACS into different countries and settings to ensure content is not lost during the translation process and minimize cross-cultural differences. Additionally, a complementary qualitative part of the SACS might be needed for specific research questions that call for a more in-depth examination. Nevertheless, this instrument should follow a standardized concept of attitudes and specifically developed questions in this regard to prevent further blurring of the underlying definition.

## 5. Conclusions

This scoping review aimed to provide an overview of the evidence of staff attitudes towards coercion, specifically since the development of the SACS, one of the most used and adapted instruments. Results from the included studies indicate that the relevance of staff attitudes in the use of coercion in mental healthcare is increasing, which raises urgent demands to be based on solid theoretical foundations. In the assessment of staff attitudes toward coercion, we found a broad variety of underlying concepts and a lack of those being reflected in the instruments used. After 15 years of using the SACS, we identified several implications to update the SACS based on existing evidence. Those include the inclusion of items addressing the emotional aspect of attitudes, as well as developing different versions of the SACS for other relevant stakeholders. Furthermore, the review highlights the need for developing a standardized procedure when adapting the SACS or other assessment instruments to different languages and settings. Those efforts in terms of methodological approach could contribute to further improvement of the research of staff attitudes towards coercion and pave the way for a considerable gain in knowledge in this area.

## Figures and Tables

**Table 1:** Overview of general information and study characteristics of all studies reviewed (*n* = 80).

	*n* (%)
Year of publication	
2008	3 (3.8)
2009–2013	16 (20.8)
2014–2018	27 (33.75)
2019–2023	34 (42.5)
Regions of origin	
Africa	2 (2.5)
Asia	28 (35.0)
Australia	9 (11.3)
Europe	40 (50.0)
South America	1 (1.3)
Study population	
Nurses	65 (81.3)
Physicians	37 (29.6)
Other professional groups	32 (40.0)
Study design	
Quantitative	67 (83.8)
Qualitative	10 (12.5)
Mixed method	3 (3.8)
Study aim	
Examination of staff’s general perception of coercion	23 (28.8)
Examination of staff attitudes towards coercion	16 (20.0)
Examination of the association between staff attitudes and other traits or possible influencing factors	13 (16.3)
Examination of the association between staff attitudes and the actual use of coercive measures	8 (10.0)
Comparison of attitudes towards coercion in staff and other groups including relatives and service users	5 (6.3)
Examination of the influence of certain interventions on staff attitudes toward coercion	7 (8.8)
Development and validation of instruments to assess staff attitudes towards coercion	8 (10.0)

**Table 2:** Overview of identified challenges in the assessment of staff attitudes.

	*n* (%)
Attitudes defined as (*n* = 22)	
(political) opinions, beliefs, and values	2 (9.1)
normative attitudes	2 (9.1)
approval to use coercion	2 (9.1)
learned predisposition (theory of reasoned action)	8 (36.4)
feelings about coercion	2 (9.1)
preferences towards specific coercive measures	1 (4.5)
(part of) ethical consideration	3 (13.6)
the perception of coercion (necessity and appropriateness)	2 (9.1)
Assessment instrument (*n* = 48)	
SACS (Staff Attitude to Coercion Scale), [25]	21 (43.8)
ACMQ (Attitudes to Containment Measures Questionnaire), [17,18]	10 (20.8)
KAPS (Knowledge, Attitude, and Practice on Seclusion), [19]	10 (20.8)
SNASS (Survey of nurses’ attitudes towards seclusion), [34]	6 (12.5)
PATS-Q (Professional Attitudes Toward Seclusion Questionnaire), [35]	2 (4.2)
SREQ (Seclusion and Restraint Experience Questionnaire) [36]	1 (2.1)
Limitations addressed in studies reviewed (*n* = 33, multiple answers possible)	
Use of self-reported questionnaires	3 (9.1)
Cross-sectional design	8 (24.2)
Bias of acceptability/social desiredness	7 (21.2)
Neglection of emotional and behavioral components of attitudes	4 (12.1)
Missing evidence on general stability or possible fluidity	3 (9.1)
Translation bias	5 (15.2)
Change in structure, mode of assessment	2 (6.1)
Lack of a gold standard for validation	1 (3.0)
Lack of generalizability of results	6 (18.2)
Difficulties of cultural and institutional influence	10 (30.3)
Missing assessment of attitudes of other stakeholders	2 (6.1)

## Data Availability

The data presented in this study that are not published in the Supplementary Materials are available on request from the corresponding author.

## Supplementary Materials

The following supporting information can be downloaded at: https://www.mdpi.com/article/10.3390/healthcare12161552/s1, File S1: Search history including all search terms and number of results for each database. File S2: Overview of data extracted from studies reviewed.
