# Supplementary material for: A Scoping Review on Staff Attitudes towards the Use of Coercion in Mental Healthcare
Source: Healthcare (Basel). 2024 Aug 6;12(16):1552. doi: 10.3390/healthcare12161552 (PMC11354183; doi:10.3390/healthcare12161552)
Supplement: Supplementary file 1 [file healthcare-12-01552-s001.zip › healthcare-3104367-supplementary.pdf]

Supplementary file 1: Search history including all search terms and number of results for each database

Database: Ovid MEDLINE(R) ALL <1946 to June 29, 2023>

Date: 03.07.2023

Number of results: 1000

#	Searches	Results
1	Restraint, Physical/	12621
2	Coercion/	5072
3	Involuntary Commitment/	72
4	Involuntary Treatment/	129
5	(seclu* or restraint* or isolation* or (solitar* adj2 confine*) or involuntary or outpatient commitment* or coerc* or ((forced or compul*) adj2 (treatment* or medication* or examination*)) or (compuls* adj2 (hospitali* or admission*)) or sectioned mental health or community treatment order or ((constant or intermittent) adj2 observation*)).tw,kf,kw.	357235
6	1 or 2 or 3 or 4 or 5	365479
7	Psychiatric Nursing/	18220
8	(Medical Staff, Hospital/ or Nurses/ or Physicians/ or Medical Staff/ or Nursing Staff/ or Nursing Staff, Hospital/ or Social Workers/ or Professional Role/) and (Mental Health Services/ or Psychiatry/ or Psychology/ or Hospitals, Psychiatric/ or Psychiatric Department Hospital/)	4050
9	(psychiatrist* or psychologist* or ((mental health or psychiat* or psychol*) adj11 (nurse* or nursing or physician* or professional* or personnel* or staff* or social worker* or therapist*)) or institutional attendant* or ((mental or psychiat*) adj11 (unit* or hospital* or ward* or department*))).tw,kw,kf.	149429
10	7 or 8 or 9	159657
11	Attitude/	53725
12	Morale/	2984
13	Morals/	18825
14	(attitude* or opinion* or moral* or ethic* or view* or perception*).tw,kw,kf.	1287239
15	11 or 12 or 13 or 14	1314765
16	6 and 10 and 15	1537
17	limit 16 to yr="2008 -Current"	1000

https://ovidsp.ovid.com/ovidweb.cgi?T=JS&NEWS=N&PAGE=main&SHAREDSEARCHID=5hPL8VXzmwSIuHiEGbFABL8sfATPTn7WTm4qZ3ywefOHMqaMsei6zbUXyKbG989g0

Database: Ovid APA PsycInfo <1806 to June Week 4 2023>

Date: 03.07.2023

Number of Results: 1333

#	Searches	Results
1	patient seclusion/	629
2	physical restraint/	2362
3	social deprivation/	1015
4	involuntary treatment/	1594
5	coercion/	2920
6	(seclu* or restraint* or isolation* or (solitar* adj2 confine*) or involuntary or outpatient commitment* or coerc* or ((forced or compul*) adj2 (treatment* or medication* or examination*)) or (compuls* adj2 (hospitali* or admission*)) or sectioned mental health or community treatment order or ((constant or intermittent) adj2 observation*)).tw.	73909
7	or/1-6	75478
8	mental health personnel/	12715
9	clinical psychologists/	3238
10	psychiatric hospital staff/	1188
11	institutional attendants/	612
12	psychiatric nurses/	3971
13	psychiatric social workers/	223
14	psychiatrists/	12813
15	(professional personnel/ or exp health personnel/ or exp therapists/) and (exp psychiatry/ or exp psychology/ or mental health/ or psychiatric hospitals/ or psychiatric units/ or psychiatric clinics/ or exp psychiatric hospitalization/ or exp mental health commitment/ or exp psychiatric hospital admission/ or psychiatric hospital discharge/ or exp mental health services/)	29434
16	(psychiatrist* or psychologist* or ((mental health or psychiat* or psychol*) adj11 (nurse* or nursing or physician* or professional* or personnel* or staff* or social worker* or therapist*)) or institutional attendant* or	249724

	((mental or psychiat*) adj11 (unit* or hospital* or ward* or department*))).tw.	
17	or/8-16	265077
18	attitudes/	31162
19	health personnel attitudes/	23109
20	psychologist attitudes/	1225
21	counselor attitudes/	2114
22	therapist attitudes/	3072
23	occupational attitudes/	2871
24	(attitude* or opinion* or moral* or ethic* or view* or perception*).tw.	1025213
25	or/18-24	1041803
26	7 and 17 and 25	2360
27	limit 26 to yr="2008 -Current"	1333

https://ovidsp.ovid.com/ovidweb.cgi?T=JS&NEWS=N&PAGE=main&SHAREDSEARCHID=376g2ZNeYByiqXaXhNDBNkq7LQ9j0qOqzSOrj8azJCcGWzpOROYgsyG6AMgdY0dx8

Database: Cinahl via EBSCOhost Research Databases

Date: 03.07.2023

Number of results: 680

#	Searches	Results
S1	MH "Patient Seclusion"	699
S2	MH "Restraint, Physical"	4,615
S3	MH "Coercion"	2,605
S4	MH "Involuntary Treatment"	80
S5	MH "Involuntary Commitment"	2,036
S6	TI ((seclu* or restraint* or isolation* or (solitar* N1 confine*) or involuntary or “outpatient commitment*” or coerc* or ((forced or compul*) N1 (treatment* or medication* or examination*)) or (compuls* N1 (hospitali* or admission*)) or “sectioned mental health” or “Community treatment order” or ((constant or intermittent) N1 observation*))) OR AB ((seclu* or restraint* or isolation* or (solitar* N1 confine*) or involuntary or “outpatient commitment*” or coerc* or ((forced or compul*) N1 (treatment* or medication* or examination*)) or (compuls* N1 (hospitali* or admission*)) or “sectioned mental health” or “Community	48,934

	treatment order” or ((constant or intermittent) N1 observation*)))	
S7	S1 OR S2 OR S3 OR S4 OR S5 OR S6	53,091
S8	MH "Psychiatrists"	3,618
S9	MH "Psychologists”	5,114
S10	MH "Psychiatric Mental Health Nurse Practitioners"	150
S11	MH "Psychiatric Nursing"	18,961
S12	(MH "Medical Staff, Hospital" OR MH "Medical Staff" OR MH "Social Workers" OR MH "Nurses" OR MH "Physicians" OR MH "Mental Health Personnel") AND (MH "Mental Health Services" OR MH "Psychiatry" OR MH "Psychology" OR MH "Psychiatric Care” OR MH "Hospitals, Psychiatric" OR MH "Psychiatric Units")	3,258
S13	TI ((psychiatrist* or psychologist* or ((“mental health” or psychiat* or psychol*) N10 (nurse* or nursing or physician* or professional* or personnel* or staff* or “social worker*” or therapist*)) or “institutional attendant*” or ((mental or psychiat*) N10 (unit* or hospital* or ward* or department*)))) OR AB ((psychiatrist* or psychologist* or ((“mental health” or psychiat* or psychol*) N10 (nurse* or nursing or physician* or professional* or personnel* or staff* or “social worker*” or therapist*)) or “institutional attendant*” or ((mental or psychiat*) N10 (unit* or hospital* or ward* or department*))))	77,471
S14	S8 OR S9 OR S10 OR S11 OR S12 OR S13	92,277
S15	MH "Attitude"	17,570
S16	MH "Morale"	1,574
S17	MH "Morals"	9,920
S18	TI ((attitude* or opinion* or moral* or ethic* or view* or perception*)) OR AB ((attitude* or opinion* or moral* or ethic* or view* or perception*))	475,715
S19	S15 OR S16 OR S17 OR S18	486,242
S20	S7 AND S14 AND S19 Limiters - Published Date: 20080101-20231231	680

Database: Web of Science via Clarivate

Date: 03.07.2023

Number of Results: 1306

#	Searches	Results

#1	TS=(seclu* or restraint* or isolation* or (solitar* NEAR/1 confine*) or involuntary or "outpatient commitment*" or coerc* or ((forced or compul*) NEAR/1 (treatment* or medication* or examination*)) or (compuls* NEAR/1 (hospitali* or admission*)) or "sectioned mental health" or "community treatment order" or ((constant or intermittent) NEAR/1 observation*))	500,587
#2	TS=(psychiatrist* or psychologist* or (("mental health" or psychiat* or psychol*) NEAR/10 (nurse* or nursing or physician* or professional* or personnel* or staff* or "social worker*" or therapist*)) or "institutional attendant*" or ((mental or psychiat*) NEAR/10 (unit* or hospital* or ward* or department*)))	138,218
#3	TS=(attitude* or opinion* or moral* or ethic* or view* or perception*)	2,512,715
#4	PY=(2008 or 2009 or 2010 or 2011 or 2012 or 2013 or 2014 or 2015 or 2016 or 2017 or 2018 or 2019 or 2020 or 2021 or 2022 or 2023)	36,606,238
#5	#1 AND #2 AND #3 AND #4	1,306

https://www.webofscience.com/wos/woscc/summary/35fc3228-13c9-48e0-aedd-22bc64af3101-9552fe23/relevance/1

Database: Google Scholar via Publish or Perish

Date: 03.07.2023

Number of results: 202

coercion|coercive|seclusion|restraints|involuntary|compulsory|psychiatrists|psychologist|”mental health”|psychiatric|psychiatry|psychological|staff|nurses|nursing|physicians|professionals|personnel attitude|opinion|moral|morale|ethical|views|perception

Supplementary File 2: Overview of data extracted from studies reviewed

Title/Author/Year	Country	Purpose	Study Design	Key Findings
Between authoritarian and dialogical approaches: Attitudes and opinions on coercion among professionals in mental health and addiction care in Norway (Aasland 2018)	Norway	To describe how different professions who work in mental health and substance abuse settings perceive the necessity for the use of different coercive measures.	Quantitative, vignette survey	There is a considerable variety in the respondents' choices among the different suggested actions, but more often towards non-coercive or dialogical interventions than not. The majority of the respondents sometimes prefer actions that are illegal, which suggests that individual opinions about coercion often overrule legislation. However, the most striking finding is the almost systematic polarity between psychologists and psychiatrists: Psychologists are less authoritative and more dialogical, while psychiatrists are more willing to apply coercion.
Correlates of psychiatric staff's attitude toward coercion and their sociodemographic characteristics (Al-Maraira 2020)	Jordan	To identify the difference in psychiatric staff attitudes toward coercive measures with their sociodemographic characteristics.	Quantitative, cross-sectional, descriptive, correlational	The relationship between the clinical unit, gender-based units, years of experience and attitude toward coercion was significant. The more years of experience in psychiatric nursing, the more positive the attitude toward coercion. Moreover, working with male psychiatric patients and in acute psychiatric units increase the risk of coercive measures use.
Psychiatric staff attitudes toward coercive measures: An experimental design (Al-Maraira 2019)	Jordan	To evaluate the effectiveness of a training program on the attitudes of psychiatric nurses toward using coercive measures.	Quantitative, experimental	After four weeks of training improving attitudes, nurses in the intervention group demonstrated significant improvements in their attitude mean scores.
Use of coercion in mental healthcare services in Nigeria: Service providers' perspective (Aluh 2023)	Nigeria	To find out how mental health professionals in Nigeria perceive coercion and what contextual factors influence their use.	Qualitative, explorative	Individual staff attitudes were also noted to influence the use of coercion. A large proportion of the professionals also felt that coercion was integral to the practice of psychiatry.
Validation of the "staff attitude toward coercion use in treatment of mentally ill patients" questionnaire in selected public psychiatric hospitals of Tehran in 2015 (Arab 2017)	Iran	To validate the "staff attitude toward the use of coercion in the treatment of mentally ill patients" questionnaire and assess their attitude in selected public psychiatric hospitals in Tehran city.	Quantitative, descriptive, analytical	Coercion as treatment and care has a higher score than coercion as an insult. the participants in the present study believed that by spending enough time, the use of coercion in treatment could be reduced (this question obtained the highest score). There were statistically significant differences between marital status and insult attitude, also between job and education variables and insult attitude and security and treatment domains.
Experience of coercion among nursing professionals in a medium-stay mental health unit: A qualitative study in Spain (Aragones-Calleja 2023)	Spain	To explore the knowledge, perception and experience of coercion among nursing staff at a rehabilitation	Qualitative, interview study	Two main themes were found: (1) therapeutic relationship and treatment in the medium stay MH unit, which included three subthemes: qualities of the professionals for building the therapeutic relationship, perceptions about the persons admitted to the MSMHU, views of the therapeutic relationship and treatment in the MSMHU; (2) Coercion at the MSMHU, comprising five

		medium-stay mental health unit in Eastern Spain.		subthemes: professional knowledge, general aspects; emotional impact of coercion; opinions; alternatives.
[Cologne Questionnaire on Attitudes Towards Coercive Measures (KEZ)] (Baar 2022)	Germany	To develop a test-theoretically funded questionnaire to assess attitudes of mental health professionals toward the use of coercion.	Mixed-methods, qualitative interview study, quantitative survey	A test-theoretical analysis led to a 39 item set with a high consistency of the overall scale (Cronbach's α =0.904) and three factors: 1) Acceptance of coercive measures without questioning (Cronbach's α = 0.797); 2) Meaningfulness and legitimation of coercive measures (Cronbach's α = 0.812); and 3) Security and order through coercive measures (Cronbach's α = 0.791). With the KEZ an instrument is available that holistically presents the various aspects of the attitudes of psychiatric staff to the use of coercion.
Nurses' attitudes towards the use of PRN psychotropic medications in acute and forensic mental health settings (Barr 2018)	Australia	To identify nurses attitudes and practice preferences in relation to PRN medication administration with consumers in a forensic and non-forensic acute mental health setting in Australia.	Quantitative, descriptive and comparative	Decision to use PRN medications in acute mental health and forensic settings remains dependent on the knowledge, skills and attitudes of individual nurses and this decision is supported by the service s medication prescribing culture. Practice differences between forensic and other acute mental health settings were identified related to the use of PRN medications to manage symptoms from nicotine, alcohol and other drug withdrawal.
Seclusion and the importance of contextual factors: An innovation project revisited (Boumans 2015)	Netherlands	To investigate whether an innovation project contributed to a change in attitudes toward seclusion and/or decision making on seclusion and/or an increase in work engagement of the nurses of an experimental ward as compared with the nurses of control wards.	Quantitative, explorative	As for attitudes toward and decision making on seclusion, changes were related to the specific organizational phase, without a differential effect between the experimental ward and the control wards.
The Heyman Survey of nursing employees' attitudes towards mechanical restraints in Slovenia (Bregar 2019)	Slovenia	To assess the attitude of Slovene psychiatric nursing professionals towards using mechanical restraint.	Quantitative, descriptive and exploratory non-experimental	Differences in the average duration of administered mechanical restraint between individual hospitals. Staff most often stated that patients felt angry when subjected to mechanical restraint. Most respondents believe mechanical restraints can be an effective therapeutic tool. Females with higher education experience statistically significantly more negative emotions and are less inclined to use mechanical restraints.
Cross-sectional study on nurses' attitudes regarding coercive measures: the importance of socio-demographic characteristics, job satisfaction, and strategies for coping with stress (Bregar 2018)	Slovenia	To research the influence of gender, age, years of service, education, working environment (closed/open ward), differences per individual psypsychiatric hospital, job satisfaction,	Quantitative, cross-sectional	Nurses attitudes towards special coercive measures are predominantly negative. The factors that explain a positive attitude were: female gender, fewer years of service, emotion-focused strategies of coping with stress, and less-threatening patient behavior.

		various strategies for coping with stress, on such attitudes among nurse practitioners in Slovenia.		
Psychiatric Nurses' Knowledge, Attitudes, and Practice Regarding Physical Restraint in China: A Multicentre Cross-Sectional Study (Chong 2023)	China	To explore the situation and influencing factors of the psychiatric nurses’ knowledge, attitudes and practices regarding physical restraint.	Quantitative, cross-sectional, descriptive	Nurses had a good level of knowledge with positive attitudes and adequate practices. They had some misunderstandings and undesirable patterns. Educational background, position, and training experience was the main factor influencing physical restraint knowledge, attitudes and practice among psychiatric nurses.
The Impact of 'Being There': Psychiatric Staff Attitudes on the Use of Restraint (Dahan 2018)	Israel	To explore differences in attitudes towards mechanical restraint in psychiatry based on level of exposure of staff members to incidences of restraint.	Quantitative, descriptive	Compared to those who were not present during restraint, staff members who were present agreed significantly less with statements indicating that restraints are humiliating and offending and agreed more with statements indicating that restraints are used primarily for security and care ($p < .05$). Among those present in incidences of restraint, staff members who physically participated in restraint agreed significantly more with statements indicating that restraints are a means for security, care and order, and less with statements indicating restraints are humiliating and offending, compared to those present but not physically participating in restraint ($p < .05$).
[Attitude of psychiatrists and psychiatry residents in Lebanon towards restraint and informed consent] (Daou 2021)	Lebanon	To study the attitude of psychiatrists and residents in psychiatry in Lebanon about restraint and informed consent.	Quantitative, cross-sectional	70% of informants did not find that restraint is commonly used in hospital practice. 92.5% would use it to counter the patient’s dangerousness, and 60% to help deliver treatment. 57.5% did not find the repeated usage of restraint as a dehumanization of care. The majority (70%) agreed with the need for temporal limitation of any restraint.
Coercive containment measures for the management of self-cutting versus general disturbed behaviour: Differences in use and attitudes among mental health nursing staff (Dickens 2022)	UK	To investigate mental health nurses’ use of and attitudes toward coercive interventions for the management of self-cutting.	Quantitative, cross-sectional	Respondents disapproved of using each coercive measure for self-cutting more than they did for disturbed behaviour except for PRN medication. Attitudes to coercive measures differed across target behaviours. Nurses who had used each measure for managing self-cutting disapproved of it less for that purpose than those who had not. Nurses who had used coercive techniques for self-cutting management had less desirable attitudes to their use.
Attitudes among stakeholders towards compulsory mental health care in Norway (Diseth 2011)	Norway	To identify prototypical attitudes and to test possible differences of attitudes between groups of stakeholders towards the use of coercion in mental health care.	Quantitative, explorative, q-method	The most widely shared attitude stated that a trusting relationship between patient and therapist is more important than the right to have an attorney. differences in attitude could in part be explained by the respondents' role in mental health care. Both psychiatrists and “somatic” physicians expressed more agreement with the present legislation than the other stakeholders.
A German Version of the Staff Attitude to Coercion Scale.	Germany	To develop and adapt the original version of the SACS into the German language	Quantitative	Based on the results, staff members could be categorized into three groups (rejecting coercion, approving coercion, or ambivalent) according to their value on the scale.

Development and Empirical Validation (Efkemann 2020)		and context. Secondly, to examine its feasibility, reliability and validity.		
Influence of mental health professionals' attitudes and personality traits on decision-making around coercion: Results from an experimental quantitative survey using case vignettes (Efkemann 2022)	Germany	To examine whether staff attitudes towards coercion influence decision-making around coercion in individual cases in mental healthcare in an experimental setting.	Quantitative, cross-sectional, case vignette study	An approving attitude towards the use of coercion reflects seeing coercion as a necessary part of psychiatric care, especially to provide security for patients and professionals. A disapproving attitude towards the use of coercion reflects seeing coercion as being offensive towards the patients.
Patient and staff perspectives on the use of seclusion (El-Badri 2008)	New Zealand	To investigate the perceptions and experiences of patients and staff on the use of seclusion in psychiatric services.	Mixed-methods, cross-sectional, inductive and systematic exploration	The use of seclusion is frequently associated with negative emotions in staff and patients alike, and it is thought to be overused. Staff and patients both attributed more negative than positive feelings to patients experience of seclusion. Both staff and patients felt that the major emotional impact of seclusion on the patient was fear.
Psychiatric Nurses' Perceptions about Physical Restraint; A Qualitative Study (Fereidooni Moghadam 2014)	Iran	To investigate the psychiatric nurses experiences of using physical restraint in the psychiatric wards of Ahvaz hospitals, southern Iran.	Qualitative content- analysis	Four categories emerged: (1) Restraint as a multi-purpose procedure, (2) Processing of physical restraint, (3) Restraint as a challenging subject and (4) The effects of restraint on the spectrum. The participants described using physical restraint as one of the main strategies to control psychiatric patients.
Development of the Japanese version of Staff Attitude to Coercion Scale (Fukasawa 2022)	Japan	To develop the Japanese version of the Staff Attitude to Coercion Scale (SACS) and clarify its psychometric properties.	Quantitative	Construct validity of the SACS Japanese version was partially confirmed. The reliability of the total scale was good. In Japan, using the subscales was not recommended, using the total scale of SACS seemed acceptable.
Staff attitudes and perceptions towards the use of coercive measures in psychiatric patients (Galbert 2023)	Israel	To examine the attitudes and perceptions regarding the use of PCMs by clinical and auxiliary workers to evaluate the level of staff willingness to reduce the use of PCMs and the level of the perceived institutional support provided after coercive intervention to staff members who participated in the event.	Quantitative, cross-sectional	A low degree of support for the use of physical coercive measures (PCM) among participants who were older, female, and more qualified psychiatric nurses, with longer duration of employment, and those who had not participated in coercive intervention in the past year. The majority of the sample reported a low willingness to reduce the use of PCMs, and a lack of institutional support after participating in a coercive event. High hospital occupancy and insufficient staffing were perceived as contributing factors to coercive incidents.

Indian nurses' Knowledge, Attitude and Practice towards use of physical restraints in psychiatric patients (Gandhi 2018)	India	To assess knowledge, attitude and practice towards use of physical restraints among nurses those working in psychiatric health care settings.	Quantitative, descriptive	Nurses had favorable attitudes as reflected in good practice of using physical restraints in psychiatric patients. Females and nurses with more than ten years of experience were found to have favorable attitudes than male nurses and nurses with less experience. A significant correlation was found between nurses’ total scores of knowledge (p<0.01), attitude (p<0.001) with their practice of using physical restraints.
Attitudes, opinions, behaviors, and emotions of the nursing staff toward patient restraint (Gelkopf 2009)	Israel	To examine nurses’ attitudes regarding the goals of restraint, the environmental conditions influencing restraint, the emotional aspects of restraint, and their beliefs about whether other staff members should participate in restraint procedures.	Quantitative, cross-sectional	The variables that influence the nursing staff’s comprehension of the goals of patient restraint are first and foremost their level of qualification, followed by the gender of the caregiver, the department where they work, the array of instruments available to the staff to cope with violence and environmental conditions. The higher the level of qualification, the more nurses considered restraint a therapeutic instrument for dealing with violence. The less qualified nurses considered patient involvement in annoying activities as an adequate cause for restraint. Nurses with lower levels of qualification view restraint with a negative connotation of humiliation, punishment, and the staff’s inability to cope with violence. Women develop more negative feelings and believe that restraints reflect the inability of the staff to cope with violence.
Perceptions of nurses working with psychiatric consumers regarding the elimination of seclusion and restraint in psychiatric inpatient settings and emergency departments: An Australian survey (Gerace 2019)	Australia	To understand the perceptions and attitudes of nurses towards containment practices, experiences in using the methods, thoughts regarding their elimination, and barriers and enablers in the elimination.	Quantitative, cross-sectional	Seclusion and restraint were viewed as necessary last-resort methods to maintain staff and consumer safety. Nurses tended to disagree that containment methods could be eliminated from practice. Seclusion was considered significantly more favorably than mechanical restraint, with the elimination of mechanical restraint seen as more of a possibility than seclusion or physical restraint. Respondents accepted that the use of these methods was deleterious to relationships with consumers. They felt that containment was used as a function of a lack of resources.
Determination of psychiatric clinic nurses' knowledge, attitudes, and practices regarding the use of physical restraints (Goktas 2018)	Turkey	To determine nurses’ knowledge, attitudes, and practices regarding the use of physical restraint in psychiatric settings.	Quantitative, descriptive	It was found that nurses’knowledge level regarding physical restraint was good, attitudes were negative, and practices were close to excellent. The mean knowledge score of nurses who did not use physical restraints (p=0.031) was statistically higher than others� The mean attitudes score of nurses whose age was between 20 and 35 (p=0.044), who were bachelors (p=0.026), and who did not use physical restraints (p=0.034) were statistically higher than others� The mean practice scores of women (p=0.005) and nurses who had over 10 years’ clinical experiences (p=0.03) were statistically higher than others.
Clinician attitude and perspective on the use of coercive measures in clinical practice from tertiary care mental health establishment - A cross-sectional study (Gowda 2019)	India	To study psychiatrists' attitudes and perspectives on the use of coercive measures in clinical practice against the background of family and patients' opinion.	Quantitative, cross-sectional	Psychiatrists perceived coercion as care, protection and safety, and as protection from dangerous situations. About 66% of psychiatrists perceived physical and chemical restraint (sedation) as necessary and acceptable in acute emergency care. One-third of the psychiatrists felt their patients lost autonomy, dignity, and the possibility of interpersonal contact. They agreed that some patients could have been treated with less restriction and fewer coercive measures.

[Use of restraint in psychiatry: Feelings of caregivers and ethical perspectives] (Guivarch 2013)	France	To study the feelings of caregivers facing restraint from an ethical perspective and identify improvement areas.	Quantitative, cross-sectional, epidemiological	The emotional experience of caregivers was rich, intense and predominantly negative type of frustration (35% of nurses; 66.7% of doctors), anger (30 and 33.3%) and lack of feeling (35 and 44.4%). The feelings of doctors and nurses were not entirely similar. For caregivers, it was ‘‘a difficult but necessary experience⍰⍰(82.75%), and ‘‘an act of care and safety⍰⍰(68.9%). All psychiatrists and almost half of the nurses (45%) said they did not feel the same when they used seclusion. Seclusion entailed a less painful experience because of its therapeutic properties. More than half of the caregivers thought that there were alternatives to restraint. They identified contexts (80%) encouraging the use of restraints, not only related to the patient the lack of resources but also institutional contexts, in particular conflicts or divisions in the health care team.
The Relationships between Attitudes toward Seclusion and Levels of Burnout, Staff Satisfaction, and Therapeutic Optimism in a District Health Service (Happell 2012)	Australia	To investigate whether staff attitudes toward seclusion were related to levels of burnout, staff satisfaction, and therapeutic optimism to determine the role of staff in decision-making about seclusion.	Quantitative, cross-sectional	Correlations between perceiving the patients as feeling punished by seclusion and intrinsic satisfaction and between patients asking to go to the seclusion room and personal accomplishment. Most correlations were small or negligible in size. Some participants raised doubts about whether it is being employed solely as a measure of last resort.
Attitudes to the use of seclusion: has contemporary mental health policy made a difference? (Happell 2010)	Australia	To determine whether seclusion reduction in government policy has been associated with a change in nurses⍰attitudes to using seclusion.	Quantitative, cross-sectional	Participants recognised the negative impact of seclusion on consumers; however, they continue to support its use, particularly in cases of threatened or actual violence to staff and other consumers. The impact of the seclusion room on consumers was viewed as significant. Most participants did not recommend changes other than painting the room for a calming effect. Demographic factors had a limited impact on attitudes (age, years of work experience and years of work with the current unit, but most were not meaningful).
Impacts of seclusion and the seclusion room: exploring the perceptions of mental health nurses in Australia (Happell 2011a)	Australia	To examine nurses⍰ perceptions of the impact of seclusion and the seclusion room on patients the changes nurses would consider in the seclusion room about characteristics of the nurses, including therapeutic optimism, job satisfaction, and burnout.	Quantitative, cross-sectional	Relationship between the three scales and attitudes to seclusion; however, therapeutic optimism had the strongest link. Participants with higher scores for optimism were more likely to acknowledge the detrimental effects of seclusion and the seclusion room and were more likely to support positive changes to the seclusion room.
Scratching beneath the surface: influencing factors on nurses' attitudes toward the use of seclusion (Happell 2011b)	Australia	To consider how factors such as therapeutic optimism, job satisfaction, and burnout might relate	Quantitative, cross-sectional	Significant relationships between attitudes toward seclusion and therapeutic optimism, job satisfaction, and burnout. Participants with higher optimism scores, high intrinsic motivation, low emotional exhaustion, and high personal accomplishment were more likely to respond negatively to the use of seclusion.

		to nurses' attitudes toward seclusion.		
Seclusion as a necessary intervention: the relationship between burnout, job satisfaction and therapeutic optimism and justification for the use of seclusion (Happell 2011c)	Australia	To investigate the relationship between burnout, job satisfaction and therapeutic optimism and justification of the use of seclusion.	Quantitative, cross-sectional survey	Most participants considered certain behaviours, particularly those involving harm to self, others or property, as appropriate reasons for using seclusion and were consistent with their perceptions of the likely practice in their unit. Association between therapeutic optimism and emotional exhaustion (burnout) and justifications for the use of seclusion. Higher optimism scores and lower scores for emotional exhaustion were significantly less likely to support using seclusion in specific situations.
Psychiatric nurses' knowledge, attitudes, and practice towards the use of physical restraints (Hasan 2019)	Saudi Arabia	To examine psychiatric nurses' knowledge and attitudes towards the use of physical restraint, as well as their practice in using it.	Quantitative, cross-sectional, descriptive, correlational	Participants had a moderate knowledge and attitude and practice in using physical restraint. Less than half reported that they recognized alternative approaches to physical restraint, and most of them did not understand the reasons for the restraint. Correlation between academic qualification and knowledge, attitudes, and practice of nurses towards physical restraint, as degree and post- basic certified nurses showed a higher knowledge score in its use.
Attitudinal variance among patients, next of kin and health care professionals towards the use of containment measures in three psychiatric hospitals in Switzerland (Hotzy 2019)	Switzerland	To compare the attitudes towards containment measures between three sites in Switzerland, which differ in their clinic traditions, policies and their actual usage of these measures.	Quantitative, cross-sectional	Substantial differences in the usage of and the attitudes towards some containment measures between the three study sites. The differences between study sites were bigger in the health care professionals' attitudes, compared to families and patients. The personnel attitudes are more associated with the clinic traditions and policies compared to patients' and their families' attitudes.
A cross cultural comparison of attitude of mental healthcare professionals towards involuntary treatment orders (Hsieh 2017)	Taiwan	To investigate East-West cultural attitudes on mental healthcare professionals towards involuntary treatment orders.	Quantitative, cross-sectional	Female respondents had higher approval ratings, agree that involuntary treatment orders were of benefit to the therapeutic relationship, assured long-term stability, and increased medication compliance. Clinicians use ITOs primarily based on risk management, regarding starting and ending an order. There are cultural differences in opinions regarding ITO's.
Staff attitudes and thoughts about the use of coercion in acute psychiatric wards (Husum 2011)	Norway	To measure attitudes towards coercion among staff in Norwegian acute psychiatric wards. To analyze differences in staff attitudes towards coercion between wards. To identify factors that influence staff attitudes towards coercion.	Quantitative, cross-sectional	A substantial part of the variance in attitudes towards coercion could be attributed to ward factors. Most of the variance could be attributed to individual staff-level factors. Staff generally had a pragmatic view of the use of coercion in the daily care of patients. Staff members tend to consider the use of coercion for caregiving. Women had a marginally lower score on the Coercion as treatment sub-scale. Staff older than 40 years considered the use of coercion to be an offence against patients more than younger staff. Staff members with a university education seemed less likely to consider coercion as an offence. Those who worked night, day and evening shifts, and those who worked day and night shifts, reported higher scores on the Coercion as treatment.

The Staff Attitude to Coercion Scale (SACS): reliability, validity and feasibility (Husum 2008)	Norway	To develop a questionnaire that could measure the diversity in staff attitudes to the use of coercion in mental health care.	Quantitative	A model with three attitudes was found in SACS based on principal component analysis and clinical considerations. The three attitudes have been named.
Mental health nurses' emotions, exposure to patient aggression, attitudes to and use of coercive measures: Cross sectional questionnaire survey (Jalil 2017)	UK	To clarify the understanding of anger in staff in relation to exposure to patient aggression, attitudes towards and actual involvement in coercion (restraint and seclusion).	Quantitative, cross sectional questionnaire survey	Nurses who reported greater exposure to a related set of aggressive behaviours, mostly verbal in nature, which seemed personally derogatory, targeted, or humiliating, also reported higher levels of anger-related provocation. Exposure to mild and severe physical aggression was unrelated to nurses�emotions. Nurses�reported anger was significantly positively correlated with their endorsement of restraint as a management technique, but not with their actual involvement in restraint episodes. Significant differences in scores related to anger and fatigue, and to fatigue and guilt, between those involved/not involved in physical restraint and in physical restraint plus seclusion respectively were detected. In regression analyses, models comprising significant variables, but not the variables themselves, predicted involvement/non-involvement in coercive measures.
The effect of psychoeducation given to psychiatry nurses on level of knowledge, attitudes, and practices regarding physical restraint: A randomized controlled study (Kavak 2019)	Turkey	To determine the effect of psychoeducation on the knowledge level, attitudes, and practices regarding physical restraint.	Quantitative, randomized controlled study	Statistically significant differences were found between the pretest and posttest total mean scores on the level of knowledge (P = .000), attitudes (P = .000), and practices (P = .000) of the nurses in the control group and the study group. No significant differences were observed in the level of knowledge, attitudes, and practices regarding the use of physical restraint in the control group, whereas, for the experimental group, which was provided psychoeducation, their level of knowledge increased and their attitude and practices regarding the use of physical restraint underwent positive improvements.
Nurses' knowledge, attitudes, and practices toward physical restraint and seclusion in an inpatients' psychiatric ward (Khalil 2017)	Saudi Arabia	To examine the of influence of nurses' knowledge, attitudes and practices on their decision to use seclusion and restraint.	Quantitative, descriptive, correlational	Moderate knowledge and attitude with strong intent to use physical restraint were found among participants. There was no significant correlation between nurses�practice, knowledge, and attitude scores. It was noted that 33.3% of the respondents preferred using both restraints and seclusion. The male gender was correlated with the use of physical restraints r = −.341, while use of seclusion had positive significant correlation with nurses�level of education r = .465, and negative correlation with other demographic background. From the current research study, it is concluded that nurses working in the Jeddah Psychiatric Hospital have a decreased level of knowledge, did not understand the patients� rights to refuse seclusion or restraints, and have little awareness about the alternative methods for seclusion and restraint, or its complications. They hold less favorable attitudes towards psychiatric patients and positive attitudes towards the use of seclusion and restraints as a method of exerting power and control over patients�behavior. However, nursing practices regarding the use of restraints and seclusion indicate that the participants use restraints in accordance with the acceptable practice. Fortunately, and promisingly, some of

				the study participants recognized a need for on ward and problem-based education, infrastructural and managerial support, as well as increasing manpower to reduce the higher use of restraint and seclusion.
The Staff Attitude to Coercion Scale (SACS) - Polish adaptation (Kiejna 2020)	Poland	To develop a Polish adaptation of the SACS.	Quantitative	After a 'think aloud' type pilot study and a language validation, the internal consistency was assessed. The Cronbach's alpha ranged from 0.57 to 0.81 in the subscales, and it was 0.82 for the total score. The best solution obtained in exploratory factor analysis was a three-factor model, almost identical to the original one, confirming the division into three subscales: coercion as offending (critical attitude), as care and security (pragmatic attitude) and as treatment (positive attitude). The psychometric characteristics of the Polish adaptation of the SACS are similar to those reported in the original version.
Attitudes towards seclusion and restraint in mental health settings: findings from a large, community-based survey of consumers, carers and mental health professionals (Kinner 2017)	Australia	To compare the attitudes of mental health service consumers, carers and mental health professionals towards seclusion and restraint in mental health settings. In particular, to explore beliefs regarding whether elimination of seclusion and restraint was desirable and possible.	Mixed-methods survey	A large majority of participants believed that seclusion and restraint practices were likely to cause harm, breach human rights, compromise trust and potentially cause or trigger past trauma. Consumers were more likely than professionals to view these practices as harmful. Many participants, particularly professionals, believed that seclusion and some forms of restraint were likely to produce some benefits, including in- creasing consumer safety, increasing the safety of staff and others and setting behavioural boundaries.
Psychiatric Nurses' Emotional and Ethical Experiences Regarding Seclusion and Restraint (Korkeila 2016)	Finland	To describe nurses emotional and ethical experiences regarding seclusion and restraint.	Quantitative, cross-section survey	The findings of this study suggest that male nurses were involved in S/R incidents more than female nurses, and at the same time, participation in ten or more S/R events during the last twelve months increased the experience of S/R being misused. Experiences related to control and duty seemed to be clearly emphasized in the nurses' responses. In the context of S/R, a lesser experience in psychiatry and also in the current ward seems to associate with positive experiences and experiences of control. Nurse Managers should therefore pay attention to these factors, for example in orientation or development discussions. However, the discussion relating to the ethics of nursing and to seclusion and restraint practices has to be continued and further explored, particularly because within the study, only half of the nurses felt that S/R violates the patient's autonomy. Without answering such an issue, patient autonomy may be compromised by continuing unclear S/R practices without agreed evidence base.
Coercion in psychiatry: A cross-sectional study on staff views and emotions (Krieger 2021)	Germany	To examine general attitudes, views and ac-companying emotions towards coercion of	Quantitative, exploratory cross-sectional online survey	Attitudes towards coercion and emotions are associated with individual staff charac-teristics (e.g. profession, work experience): Experienced staff members were most critical of coercion, whreas nurses rated coercion significantly more positively than other staff. Majority experienced compas- sion; about half felt

		different occupational groups in psychiatry.		helplessness, grief or anxiety. Almost 20% felt a sense of power. Nurses felt the most desperation.
Coercion in a locked psychiatric ward: Perspectives of patients and staff (Larsen 2014)	Norway	To investigate how patients and staff in a Norwegian locked psychiatric ward experience coercion.	Qualitative, participants observation and interviews	The participants experienced coercion in different ways. Patients often felt inferior. Many of the staff felt guilty for violating patients’ dignity, although they ascribed responsibility for their actions to the ‘‘system.’’ The main themes are (1) corrections and house rules, (2) coercion is perceived as necessary, (3) the significance of material surroundings, and (4) being treated as a human being.
A Bayesian network model to identify the associations between the use of seclusion in psychiatric care and nursing managers' attitudes towards containment methods (Laukkanen 2021)	Finland	To describe the associations between the use of seclusion and psychiatric nursing managers' attitudes towards containment methods.	Quantitative, cross-sectional descriptive	Nursing managers' age and their attitudes towards containment methods were related to the use of seclusion. Especially nursing managers' negative perceptions of seclusion were associated with less use of seclusion, and seclusion was used more often on wards with nursing managers who were older than the average.
Psychiatric nursing managers' attitudes towards containment methods in psychiatric inpatient care (Laukkanen 2020)	Finland	To examine psychiatric nursing managers' attitudes towards containment methods.	Quantitative, descriptive, cross-sectional	Psychiatric nursing managers had the most negative attitude towards net bed and mechanical restraint, and the most positive attitudes towards pro re nata medication and intermittent observation. A few associations were discovered be- tween attitudes and background variables such as gender and number of employees.
The Knowledge, Practice and Attitudes of Nurses Regarding Physical Restraint: Survey Results from Psychiatric Inpatient Settings (Lee 2021)	China	To describe knowledge, practices and attitudes about physical restraint.	Quantitative survey	In general, nurses had good restraint-related knowledge with satisfactory attitudes and practices, although their knowledge levels, attitudes, and practices regarding restraint varied. Having a higher age, seniority, and education level contributed to a higher restraint-related knowledge level. Male nurses demonstrated more desirable practices (i.e., care of restrained patients), while nurses with a higher education level were more likely to avoid restraint. Nurses’ restraint-related knowledge positively correlated with restraint practices. Although nurses’ knowledge levels, attitudes, and practices regarding restraint were found to be satisfactory, more training efforts should focus on young nurses working in psychiatric settings with less work experience and lower education levels.
Measuring Staff Attitudes to Coercion in Poland (Lickiewicz 2021)	Poland	To examine the factor structure of the Polish version of SACS, to examine the internal consistency of these factors, and to examine the reliability and face validity of SACS. To compare attitudes toward coercion between Polish nurses and psychiatrists,	Quantitative, explorative	The subscale Coercion as Treatment was rated significantly higher by nurses than by psychiatrists, but there was no difference for the two other subscales. There was no significant association between the General Self-Efficacy Scale and any of the SACS subscales. The biggest differences in attitudes toward forms of coercion was noted between Poland and Germany: Polish medical personnel see coercion as a part of the treatment and an acceptable solution in dealing with aggressive behaviors, as opposite to German respondents.

		and to examine if there was any association between self-efficacy and attitudes toward coercion.		
Psychiatric Nurses' Attitude and Practice toward Physical Restraint (Mahmoud 2017)	Sudan	To assess psychiatric nurses' attitude and practice toward physical restraint.	Quantitative, descriptive	There were insignificant differences between attitudes and practices in relation to nurses' sex, level of education, years of experience and work place. Moreover, a positive significant correlation was found between nurses' total attitude scores, and practices regarding use of physical restraint. “Psychiatric nurses have positive attitude and adequate practice toward using physical restraints as an [alternative management for aggressive psychiatric patients and not for all the patients]”. The study recommended that it is important for psychiatric nurses to acknowledge that physical restraints should be implemented as the last resort. That psychiatric nurses either have or do not have the knowledge on the application of how physical restraints should be implemented as the last resort is not at all addressed within the body of this work.
Seclusion as a necessary vs. an appropriate intervention: a vignette study among mental health nurses (Mann-Poll 2015)	Netherlands	To investigate mental health nurses' perspectives on necessity and appropriateness of seclusion.	Quantitative, vignette study	Although seclusion was scored significantly higher on necessity, factors of influence and underpinning patterns were shown largely the same. According to the theory of Festinger, focusing on a greater discrepancy between necessity and appropriateness is seen as a necessary condition to achieve a reduction in the use of seclusion. The t-test resulted in a significantly higher score on necessity than on appropriateness. Differences between both scores could be explained for 32% by a combination of nurse characteristics and vignette variables. Necessity and appropriateness were found to be strongly associated with each other, showing that underpinning patterns were largely the same.
Professionals' attitudes after a seclusion reduction program: anything changed? (Mann-Poll 2013)	Netherlands	To determine changes in professional attitudes after a seclusion reduction program.	Quantitative, pre-post design	After the program, professionals scored significantly higher on ‘ethics’ and ‘more care’. As expected, no change occurred on ‘reasons’ for the use of seclusion. In addition, no significant changes were found on ‘confidence’, ‘better care’ and ‘other care’. Significant changes in professional attitudes concerning the ethics of using seclusion and involving issues of more care were observed after a seclusion reduction program. Mental health professionals moved in the direction of ‘transformers’, indicating an increased criticism of the practice of seclusion and increased willingness to change their own use of seclusion.
Experience of mental health nurses regarding mechanical restraint in patients with psychomotor agitation: A qualitative study (Manzano-Bort 2022)	Spain	To explore MH nurses' experiences during the management and care of patients with psychomotor agitation and factors influencing the decision to use mechanical restraint.	Qualitative, interview study	Four themes emerged from the analysis: 1) Nurses’ perceptions of restraint methods, 2) Factors influencing decision-making, 3) Consequences for professionals of the use of mechanical restraint and 4) Alternatives to mechanical restraint. The present study demonstrates that, despite the general negative perception and the consequences that MR use have for patients and professionals, it continues to be a measure used in MH for managing psychomotor agitation when other means have failed, in an attempt to preserve the safety of one or both parties.

Staff's normative attitudes towards coercion: the role of moral doubt and professional context-a cross-sectional survey study (Molewijk 2017)	Norway	To examine professionals normative attitude towards coercion in relation to moral doubt.	Quantitative, cross-sectional survey	Descriptive analyses showed that in general that the respondents a) were not so sure whether coercion should be seen as offending, b) agreed with the viewpoint that coercion is needed for care and security, and c) slightly disagreed that coercion could be seen as treatment. Staff did not report high rates of moral doubt related to the use of coercion, although most of them agreed there will never be a single answer to the question ‘What is the right thing to do? Bivariate analyses showed that the more they experienced general moral doubt and relative doubt, the more one thought that coercion is offending. Especially psychologists were critical towards coercion. It was found significant differences among ward types. Respondents with decisional responsibility for coercion and leadership responsibility saw coercion less as treatment. Frequent experience with coercion was related to seeing coercion more as care and security.
Restraint and seclusion: a distressing treatment option? (Moran 2009)	Ireland	To explore emotions and feelings of nurses in response to coercive interventions (restraint, seclusion).	Qualitative, focus group study	The data were analysed using qualitative interpretive analysis. Three themes were created consisting of: (1) the last resort – restraint and seclusion; (2) emotional distress; and (3) suppressing unpleasant emotions. It is suggested that the nursesexperience of restraint and seclusion created a dynamic movement between the release and suppression of distressing emotions. The oscillatory characteristics embedded within the nursesemotional responses were reminiscent of a model of suffering developed by Morse in 2001. Consequently, this model is incorporated throughout the discussion of the findings to provide a more in-depth description of the emotional distress experienced by the nurses in the study. The findings illustrated that the nurses in the study experienced distressing emotions in response to restraint and seclusion. Therefore, they suppressed their emotions to get through these interventions. This dynamic movement between the release and suppression of emotions depicted Morses (2001) model of suffering. Morse indicates that one must fully experience emotional distress in order to overcome suffering. However, the nurses seemed to continually suppress their unpleasant emotions, which culminated in their emotional withdrawal from the client. In turn, this hindered the nursesability to communicate and relate with clients.
Mental health professionals' feelings and attitudes towards coercion (Morandi 2021)	Switzerland	To explore mental health professionals' feelings and attitudes towards coercion and sociodemographic and professional characteristics associated with them.	Quantitative, online survey	Even if a large number of the informants considered coercion a violation of fundamental rights, an important percentage of them agreed that coercion was nevertheless indis pensable in psychiatry and beneficial to the patients. ESEM revealed that professionalsfeelings and attitudes towards coercion could be described by four main dimensions labelled “Internal pressure”, “Emotional impact”, “External pressure” and “Relational involvement”. The personal as well as the professional proximity with people suffering from mental disorders influences professionalsfeeling and attitudes towards coercion. As voices recommend the end of coercion in psychiatry and despite the lack of scientific evidence, many mental health professionals remain convinced that it is a requisite tool beneficial to the patients. Clinical approaches that enhance shared

				decision making and give the opportunity to patients and professionals to share their experience and feelings towards coercion and thus alleviate stress among them should be fostered and developed.
Optimistic recovery expectations are associated with critical attitudes toward coercion among mental health professionals (Motteli 2020)	Switzerland	To examine individual and workplace characteristics associated with the attitudes of MHP toward coercion including recovery orientation.	Quantitative, cross-sectional survey	Overall, 73.6% of the professionals were critical of the use of coercion, whereas 5.5% had positive attitudes toward coercive measures. Participants with more optimistic recovery expectations, other than nurses, and those working on open wards held more critical attitudes toward coercion ($p < 0.05$). Wards characterized by less coercive measures, lower bed occupancy, fewer involuntary admissions and substance-use disorders were associated with more critical attitudes ($p < 0.05$). We conclude that training of mental health professionals—especially nurses and those who work on closed wards—in recovery orientation may help promote critical attitudes toward coercion and may reduce coercive measures.
Patients' Perspectives on and Nurses' Attitudes toward the Use of Restraint/Seclusion in a Turkish Population (Okanli 2016)	Turkey	To determine patients' perspectives on and nurses' attitudes to the use of seclusion/ restraint.	Qualitative, cross-sectional interview study	Looking at the knowledge and attitudes toward the use of restraint/seclusion, many nurses did not want restraint/seclusion to be prohibited. They were upset when a patient was restrained and regarded patients aggressive behavior (against themselves, personnel, furniture) as the cause of the restraint.
Nurses' attitudes towards professional containment methods used in psychiatric wards and perceptions of aggression in Turkey (Ozcan 2015)	Turkey	To determine nurses' attitudes towards professional containment methods and explore the relationship between these attitudes and their perception of aggression.	Quantitative, descriptive, cross-sectional	While pro re nata medication was used commonly, time-out was infrequently used in the wards. Intermittent observation, pro re nata medication and containment in the psychiatric intensive care unit were the most approved methods. The use of net beds was the least approved method. Nurses who perceive aggression as dysfunctional/undesirable are more likely to approve compulsory intramuscular medication and mechanical restraint. These results showed that nurses perception of aggression is an important factor influencing the choice of a professional containment method.
Acceptability and use of coercive methods across differing service configurations with and without seclusion and/or psychiatric intensive care units (Pettit 2017)	UK	To compare different service configurations regarding acceptability of containment methods.	Quantitative, cross-sectional	In service configurations with access to seclusion, staff rated seclusion as more acceptable and reported greater use of it. Psychiatric intensive care unit acceptability and use were not associated with its provision. Where there was no access to seclusion, staff were slower to initiate restraint. There was no relationship between acceptability of manual restraint and its initiation.
A recovery-oriented approach for an acute psychiatric ward: is it feasible and how does it affect staff satisfaction? (Rabenschlag 2014)	Switzerland	To evaluate professionals' attitude towards recovery and coercion in relation to satisfaction with work circumstances and ward atmosphere.	Quantitative, longitudinal study	The members of the intervention ward ($n = 17$) did not differ from the control group ($n = 21$), except that control group members were younger. The recovery-orientation of the study ward (ROSE questionnaire) increased significantly (alpha level = 0.05) from study begin to study end ($p = 0.003$), and compared to the control group ($p = 0.002$). The attitudes towards coercion did not change significantly in the intervention group, but did so compared to the control group. The contentedness (GMI) and the satisfaction with working conditions (ABB) of the intervention group members compared to control group was significantly higher (GMI: $p = 0.004$, ABB subscale working conditions: $p = 0.043$, satisfaction: $p = 0.023$). The study indicates that recovery-oriented principles can be

				implemented even in an acute admission ward, increasing team satisfaction with work, while attitudes towards coercion did not change significantly within this single-unit project.
Staff and caregiver attitude to coercion in India (Raveesh 2016)	India	To study sociodemographic correlates of psychiatrists and caregiver attitudes toward coercion including comparison of both.	Quantitative, cross-sectional online survey	A total of 210 psychiatrists and 210 caregivers participated in the study. Both groups agreed that coercion was related to scarce resources, security concerns, and harm reduction. Both groups agreed that coercion is necessary, but not as treatment. Older caregivers and male experienced psychiatrists considered coercion related to scarce resources to violate patient integrity. All participants considered coercion necessary for protection in dangerous situations. Professionals and caregivers significantly disagreed on most items. The reliability of the SACS was reasonable to good among the psychiatrists group, but not in the caregiver group (alpha 0.58 vs. 0.07).
Comparing Attitudes to Containment Measures of Patients, Health Care Professionals and Next of Kin (Reisch 2018)	Switzerland	To highlight the area of conflict regarding differing attitudes towards containment measures between patients, health care professionals and next of kin.	Quantitative, cross-sectional survey	In general, HCPs rated the coercive measures as more acceptable than did NOK and patients. The largest discrepancy in the ratings was found in regard to the application of coercive intramuscular injection of medication (effect size: 1.0 HCP vs. patients). However, the ratings by NOK were significantly closer to the patients ratings compared to patients and HCP. The only exception was the acceptance of treatment in a closed acute psychiatric ward, which was deemed significantly more acceptable by NOK than by patients. Also, patients who had experienced coercive measures themselves more strongly refused other measures.
Nurses' knowledge, attitude and practices on use of restraints at State Mental health care setting: An impact of in-service education programme (Rentala 2021)	India	To determine the effectiveness of short-term in-service education program in improving nurse's knowledge, attitude and self-reported practices related to physical restraint.	Quantitative, quasi-experimental study	Of the 52 nurses who participated in the study, 52% were male, 58.5% had a baccalaureate degree. The mean age of respondents was 33.3 years; the mean work experience was 6.7 years. The findings of the study revealed that the mean scores on the knowledge regarding physical restraints increased after the in-service education from 6.4 to 8.2 (p<0.001). The mean attitude scores improved from 18.5 to 23.1 (p<0.001). There was a significant difference in mean practice scores between pre and post-intervention phases (23.7 versus 25.4; p<0.001). There was a significant correlation between post-test knowledge, attitude and practice scores.
[Defining coercion and mental health care provided by nurses: a qualitative approach] (Serrano 2018)	Spain	To explore and expose the perceptions and expectations of nurses in relation to coercion and boundaries with professional values in the field of mental health care.	Qualitative, focus group study	The thematic categories identified are: “Coercion: context of discovery and context of justification”, “Human care, vulnerability and coercion in mental health” and “Strengths and weaknesses of professional care”.
Psychiatrists' attitudes towards the procedure of involuntary	China	To examine psychiatrists' attitudes towards seeking involuntary admission.	Quantitative, cross-sectional survey	Some psychiatrists in the CPA had several arbitrary attitudes towards the process of admission. Females, aged under 35, with a low education level and a low position in the institution showed stricter attitudes in the procedure of

admission to mental hospitals in China (Shao 2012)				involuntary admission. Areas with mental health legislation showed significant positive relationships with stricter attitudes.
Compulsory Interventions in Severe and Persistent Mental Illness: A Survey on Attitudes Among Psychiatrists in Switzerland (Stoll 2021)	Switzerland	To explore potential conflicts between the ethical goal of respecting patients' autonomy on the one hand and beneficence as well as paternalism on the other hand in patients with SPMI.	Quantitative, cross-sectional survey	Out of 1,311 contacted psychiatrists, 457 (34.9%) returned the completed survey. In general, 91.0% of psychiatrists found it important or very important to respect SPMI patients autonomy in decision making. However, based on three different clinical case vignettes, 36.8% of psychiatrists would act against the wishes of the patient with severe and persistent schizophrenia, 34.1% against the wishes of the patient with severe and persistent depression, and 21.1% against the wishes of the patient with severe and persistent anorexia nervosa, although all patients were stated to have preserved decision-making capacity. With regard to the case vignettes, 41.1% considered compulsory interventions leading to a temporary reduction of quality of life acceptable in the patient with severe and persistent schizophrenia, 39.4% in the patient with severe and persistent depression, and 25.6% in the patient with severe and persistent anorexia nervosa, although it was stated in all three case vignettes that two independent experts ascribed the patients decision-making capacity regarding their illness and further treatment.
A two-center pilot study on the effects of clinical ethics support on coercive measures in psychiatry (Stoll 2022)	Switzerland	To assess the effect of CES on coercion in psychiatry and on moral skills and attitudes of healthcare professionals.	Quantitative, pre-post intervention study	After implementation of MCD, formal coercion was less frequent (particularly seclusion, small effect size; 9.6 vs. 16.7%, p=.034, Cramér V=.105) and less intense (particularly mechanical restraint, large effect size; 86.8±45.3 vs. 14.5±12.1 h, exact p=.019, r=-.74), and approval for coercive measures among healthcare practitioners was lower when controlling for the number of MCD sessions attended.
Attitude of young psychiatrists toward coercive measures in psychiatry: a case vignette study in Japan (Tateno 2010)	Japan	To investigate Japanese psychiatrist's attitudes about emergency interventions by focusing on involuntary treatments and possibilities of minimizing psychiatric coercive measures.	Quantitative, case vignette study	There was general agreement among the study subjects that the case should be admitted to a hospital (8.91 ± 0.3) and secluded (8.43 ± 1.0). The estimated length of hospitalization was 13.53 ± 6.4 weeks. Regarding the likelihood of prescribing restraint, results showed great diversity (5.14 ± 2.5 on 9-point scale); psychiatrists working at general hospitals scored significantly higher (6.25 ± 2.5) than those working at university hospitals (5.02 ± 2.3) or psychiatric hospitals (4.15 ± 2.6). A two-group comparison of the length of inpatient care revealed a significant difference between those psychiatrists who scored 1-3 (n = 55, 14.22 ± 7.4) and those who scored 7-9 (n = 62, 12.22 ± 4.0) regarding the need to use restraint.
[Opinions of Nurses and Physicians Working Psychiatry Clinic on Patient Restraint Methods] (Ucun 2015)	Turkey	To determine the opinions of nurses and physicians working in psychiatry about the use of patient restraint methods.	Qualitative, descriptive survey	There were significant differences between the opinions of the nurses and the physicians in the choices about physical and chemical restraint methods. In addition to this, the importance of the education in the usage of the restraint was emphasized in both occupations. While each profession group had the similar responses on the legal and ethical matters in the usage of the restraint, it was observed that they were abstained on the approval to treat patients.

Professionals' attitudes toward reducing restraint: the case of seclusion in the Netherlands (Van Doeselaar 2008)	Netherlands	To investigate professionals' attitudes toward and experiences with constraints.	Quantitative survey (questionnaire)	The more professionals were personally involved in seclusion, the more they believed in it. Three types of professionals were identified: Transformers, Doubters and Maintainers. More than half of the psychiatrists (56%) belonged to the type of maintainers. Nurses were more divided.
The Role of Implicit and Explicit Staff Attitudes in the Use of Coercive Measures in Psychiatry (Vandamme 2021)	Germany	To investigate implicit staff attitudes and comparison of explicit and implicit attitudes.	Quantitative, cross-sectional	Results showed that there was no association between staff's implicit and explicit attitudes toward coercion, and neither measure was correlated with the local frequency of coercive measures. ANOVAs showed a significant difference of the GNAT result for the factor gender ($F = 9.32$, $p = 0.003$), demonstrating a higher tendency to justify coercion among female staff members ($M = -0.23$, $SD = \pm0.35$) compared to their male colleagues ($M = -0.41$, $SD = \pm0.31$). For the SACS, a significant difference was found for the factor profession ($F = 7.58$, $p = 0.007$), with nurses ($M = 2.79$, $SD = \pm1.40$) showing a more positive attitude to the use of coercion than psychiatrists ($M = 2.15$, $SD = \pm1.11$). No significant associations were found regarding the extent of professional experience. Results indicate a complex interaction between implicit and explicit decision-making processes dependent on specific contexts.
Physical and mechanical restraint in psychiatric units: Perceptions and experiences of nursing staff (Vedana 2018)	Brazil	To understand experiences and perceptions of nursing staff about physical and mechanical restraint in psychiatric units.	Qualitative interview study	Physical restraint was considered unpleasant, challenging, risky, and associated with dilemmas and conflicts. The nursing staff was often exposed to the risks and injuries related to restraint. Professionals sought strategies to reduce restraint-related damages, but still considered it necessary due to the lack of effective options to control aggressive behavior.
Psychometric properties of the Italian version of the staff attitude to coercion scale: an exploratory factor analysis (Venturini 2023)	Italy	To explore the factorial structure of the Italian SACS version.	Quantitative, online survey	Results confirmed the three-factor solution of the original version for the Italian version of the SACS, though three items loaded on different factors, compared to the original. The three extracted factors, explained 41% of total variance, and were labeled similarly to the original scale and according to their respective item content, i.e., Factor 1 “Coercion as offending” (items: 3, 13, 14, and 15), Factor 2 “Coercion as care and security” (items: 1, 2, 4, 5, 7, 8, and 9), and Factor 3 “Coercion as treatment” (items: 6, 10, 11, and 12). The internal consistency of the three-factor model of the Italian version of the SACS was assessed through Cronbach's α and yielded acceptable indexes, ranging from 0.64 to 0.77.
Approval ratings of inpatient coercive interventions in a national sample of mental health service users and staff in England (Whittington 2009)	UK	To compare approval ratings of staff and service users regarding coercive measures.	Quantitative, cross-sectional survey	Service users and staff strongly disapproved of net beds and mechanical restraint. The three methods that received the most approval by the service user group were intermittent observation, time out, and PRN (as needed) medication; for the staff group, the three methods that were most approved of were transfer to a psychiatric intensive care unit, PRN medication, and observation. Male staff, older service users, and staff who had been involved in implementing coercion expressed greater approval of coercive measures.
Professional values and attitude of psychiatric social workers toward	Taiwan	To identify attitudes toward involuntary hospitalization	Quantitative survey (questionnaires)	Most considered involuntary hospitalization as a means providing care and security, and supported its use. Most favored patient rights to a good environment and daily life over their right to refuse treatment and the right to

involuntary hospitalization of psychiatric patients (Wu 2013)		of psychiatric social workers.		make legal decisions. Gender, educational level, and working experience at psychiatric facilities were found to be associated with attitudes toward coercion and the rights of involuntarily hospitalized patients. Multivariate regression revealed a social worker’s holding a view that coercive hospitalization as offensive or therapeutic could predict a higher regard for the human rights of psychiatric patients.
Attitudes to coercion at two Norwegian psychiatric units (Wynn 2011)	Norway	To examine attitudes to the use of coercion in psychiatric emergencies.	Quantitative survey	There was only a limited degree of variance in how staff at the different units and various groups of staff responded. Staff were more likely to favor a highly restrictive intervention when the patients were physically violent. Male staff and unskilled staff were significantly more prone to choosing a highly restrictive intervention. Our hypothesis was confirmed, as there was a limited degree of variance in staff’s responses with respect to degree of restrictiveness. The study supported the idea that a range of different interventions are used in emergency situations.
Attitude and practice toward physical restraint among psychiatric nurses in Guangdong, China: a cross-sectional survey (Ye 2021)	China	To explore the correlation between attitude and practice toward physical restrain in psychiatric settings. The specific objectives are to (1) identify nurses’ attitude toward PR in psychiatric settings; (2) assess nurses’ practice toward PR in psychiatric settings; and (3) evaluate the correlation between the attitude and practice of nurses toward PR in psychiatric settings.	Quantitative, descriptive, correlational	The nurses’ responses showed neutral attitude and a moderate level of practice regarding the use of PR. Factors such as age, gender, marital status, professional position, nightshift, and the frequency of training programs on PR showed a significant association with nurses’ attitude and practice concerning PR ($P < 0.05$). Nurses with a largely negative attitude toward PR were more likely to use it ($OR = 1.91$, $P < 0.001$).
The effect of empathy skills of psychiatric nurses on their attitudes and practices towards the use of physical restraint (Yyldirm Üsenmez 2021)	Turkey	To evaluate the effect of empathy skills of psychiatric nurses on their attitudes and practices towards the use of physical restraint.	Quantitative, cross-sectional, descriptive, correlational	The total mean ESS score was found to be 152.34 ± 24.44, indicating moderate empathy skills. Mean KAPS-PR scores were 29.54 ± 4.43 for the attitude scale and 33.94 ± 3.26 for the practice scale, indicating appropriate attitudes and ideal practices related to physical restraint, respectively. ESS score was significantly higher among nurses with higher educational levels ($p = .001$) and weakly correlated with KAPS-PR attitude score ($r = .25$; $p < .05$).
